# Systematic Study of the Effects of High Shear Granulation Parameters on Process Yield, Granule Size, and Shape by Dynamic Image Analysis

**DOI:** 10.3390/pharmaceutics13111894

**Published:** 2021-11-08

**Authors:** Oliver Macho, Ľudmila Gabrišová, Peter Peciar, Martin Juriga, Róbert Kubinec, Pavol Rajniak, Petra Svačinová, Tereza Vařilová, Zdenka Šklubalová

**Affiliations:** 1 Institute of Process Engineering, Faculty of Mechanical Engineering, Slovak University of Technology in Bratislava, Námestie Slobody 17, 812 31 Bratislava, Slovakia; ludmila.gabrisova@stuba.sk (Ľ.G.); peter.peciar@stuba.sk (P.P.); martin.juriga@stuba.sk (M.J.); 2 Department of Analytical Chemistry, Faculty of Natural Sciences, Comenius University in Bratislava, Ilkovičova 6, 842 15 Bratislava 4, Slovakia; robert.kubinec@uniba.sk; 3 Department of Chemical and Biochemical Engineering, Faculty of Chemical and Food Technology, Slovak University of Technology in Bratislava, Radlinského 9, 812 37 Bratislava, Slovakia; pavol.rajniak@stuba.sk; 4 Department of Pharmaceutical Technology, Faculty of Pharmacy in Hradec Králové, Charles University, Ak. Heyrovského 1203, 500 05 Hradec Králové, Czech Republic; svacp3aa@faf.cuni.cz (P.S.); varilovat@faf.cuni.cz (T.V.); sklubalova@faf.cuni.cz (Z.Š.)

**Keywords:** dynamic image analysis, high shear granulation, liquid to solid ratio, granules

## Abstract

The aim of the work was to analyze the influence of process parameters of high shear granulation on the process yield and on the morphology of granules on the basis of dynamic image analysis. The amount of added granulation liquid had a significant effect on all monitored granulometric parameters and caused significant changes in the yield of the process. In regard of the shape, the most spherical granules with the smoothest surface were formed at a liquid to solid ratio of ≈1. The smallest granules were formed at an impeller speed of 700 rpm, but the granules formed at 500 rpm showed both the most desirable shape and the highest process yield. Variation in the shape factors relied not only on the process parameters, but also on the area equivalent diameter of the individual granules in the batch. A linear relationship was found between the amount of granulation liquid and the compressibility of the granules. Using response surface methodology, models for predicting the size of granules and process yield related to the amount of added liquid and the impeller speed were generated, on the basis of which the size of granules and yield can be determined with great accuracy.

## Highlights:

- Dynamic image analysis was used to analyze the morphology of high shear granules;
- The granules were investigated for size, shape, and surface roughness;
- A small change in liquid content caused changes in granule shape and process yield;
- The amount of granulation liquid had a significant effect on all parameters;
- A model for predicting the granule size was created from the experimental data.

## 1. Introduction

A wet granulation process is predominantly used in the pharmaceutical industry to manufacture tablets, which are the most widely used dosage form. The high shear wet granulation (HSWG) represents the most common method, however, fluid bed granulation (FBG) process is recently also frequent [1]. HSWG is a process of size enlargement of primary particles joined together by agitation and a liquid binder. Major advantages of this technique include: a shorter processing time, a greater densification of granules, a narrow range of operating conditions, a lower granulating fluid requirement, better predictability of the granulation end-point and better reproducibility [2]. Other benefits of HSWG include: reduced dustiness which minimizes losses, inhalation and explosion risks, improved flow and handling, controlled dissolution rates and co-mixing of particles which would otherwise segregate during handling [3]. HSWG is considered a complicated and multivariate pharmaceutical process that is influenced by a large number of variables derived from equipment, formulations, and processes [4]. The process parameters of HSWG are impeller speed, chopper speed, liquid-to-solid ratio, liquid addition rate, massing time, drying time, and drying temperature [5]. Wet massing time can also influence content uniformity in HSWG [6].

HSWG has three rate processes: wetting and nucleation, consolidation and growth, and attrition and breakage [7]. When liquid is added by spraying, the spray droplets land on the powder surface and penetrate into the pores, forming a nucleus granule [8]. During the wetting process, four states of saturation of powder by liquid may occur: pendular, funicular, capillary, and droplet state [9]. High-speed granulators are used extensively in the pharmaceutical industry because they are capable of producing granules that are small and dense, making them ideal for blending and tableting [10]. Granules formed by HSWG have a smoother and denser surface because they are subjected to an intensive consolidation process [11]. They have a narrower particle size distribution than twin-screw granules [12]. Regular particles with a smooth surface have generally better flow properties because of the fewer contact points between the particles [13]. 

Particle morphology refers to the size, shape, and surface roughness of particles [14]. There are several methods for determining the shape and size of granules based on the principle of image analysis. One such method is dynamic image analysis (DIA), in which particles are captured in motion. DIA offers numerous advantages because it does not need the density of materials for particle size distribution (PSD), it provides a continuous PSD curve, and it is fast and easy to perform. DIA, unlike other techniques, can report different size parameters as it processes the image of particles [15]. Several authors have experience with the use of DIA for different types of materials: pharmaceutical excipients [16,17,18,19], minitablets [20], tailings [21], talc [22], concrete aggregates [23], sediments [24], volcanic ash [25], calcite [26], and coal [27,28]. DIA data on particle shape, size, and distribution can be used to predict the flow and packing behavior of granular materials [29]. De Simone et al. [30] optimized the low shear granulation process of hydroxypropyl methylcellulose using DIA. Kumar et al. [31] and Madarász et al. [32] used DIA to analyze the size and shape of granules in the twin screw granulation process. 

In our work, we focused on the processing of the commonly used pharmaceutical excipient microcrystalline cellulose (MCC) by HSWG, in a wide range of liquid to solid (L/S) ratios at different impeller speeds. A formulation containing MCC requires a greater amount of water for granulation because MCC is a water-insoluble excipient having a high water-holding capacity [33]. Shi et al. [34] found that when MCC powders were processed with 35% or less water, the predominant process was wetting. Nucleation processes commenced between 35% and 45% of granulation water. Osei-Yeboah et al. [35] set the upper limit of water-holding capacity of Avicel PH101 at 135% without forming slurry. Methods of adding granulation liquid [36], its viscosity [37], but also the nozzle type [38] affect the shape of the granules formed. Nalleso et al. [39] studied the kinetic of HSWG of MCC by texture analysis during the addition of granulating liquid with different flow rates. They found that the early stages of the process (nucleation and growth) were strongly influenced by the binder flow rate. In addition, the initial moisture content can significantly affect the manufacturability of MCC granules by HSWG [40]. Impeller speed affects the size distribution and the granule size, but it also has an influence on the granules’ structure and shape. This affects the end-use properties of the granules such as the dissolution rate, compaction, and hardness during handling [41]. 

Several authors have investigated various morphological parameters for the characterization of particle shape [42,43,44,45,46,47]. Xiu et al. [48] pointed out that the flow properties of different types of MCC are directly influenced by fractal dimension and circularity. However, Almeida-Prieto et al. [49] have shown that the shape of the granules cannot be characterized correctly with only one shape factor. To fill the literature gap, the aim of our work is to systematically analyze the influence of selected HSWG process parameters on the granule morphology and process yield. Using the DIA method on a PartAn 3D device, the shape parameters sphericity, aspect ratio, roundness, and concavity will be monitored at different L/S ratios and impeller speeds; selected samples of granules will be analyzed using an electron microscope. Employing response surface methodology, the possibility to reach an acceptable model for prediction of the size of the granules will be investigated.

## 2. Material and Methods

All measurements and manipulations were carried out at a controlled ambient temperature of 24.0 ± 2.0 °C and relative air humidity 40.0 ± 12.0% analyzed by hygrometer E5005 (Emos, Prerov, Czech Republic).

### 2.1. Experimental Material

Avicel^®^ PH 101—microcrystalline cellulose (FMC Biopolymer, Cork, Ireland), a common pharmaceutical excipient obtained from the IMCD Czech Republic, was used in the experiments. This material is regularly used in the pharmaceutical industry as a filler or binder in oral dosage forms. Information about the true density (1580 kg/m^3^) and the bulk density (333 kg/m^3^) was provided by the manufacturer. An aqueous solution of polyvinylpyrrolidone powder K30 (BioChemica, Billingham, UK) was used as a granulation binder. The polyvinylpyrrolidone was prepared as a 2% *w*/*w* aqueous solution in distilled water at a temperature of 38 °C by a Stuart 162 magnetic stirrer (Stuart Equipment, Staffordshire, UK) until the powder particles were completely until the powder particles were completely dissolved.

### 2.2. Particle Size Analysis

The particle size and size distribution were analyzed using a Malvern Mastersizer 3000 Laser Diffraction Analyzer (Malvern Panalytical, Malvern, UK). Characteristic dimensions of the experimental material were assessed volumetrically, and samples were analyzed by the dry cell method. The experiment was repeated three times. The results were then interpreted as an average from three measurements. Measurements were performed using an Aero S dry powder dispersion unit. The polystyrene latex standard was used for calibration according to ISO 13320:2009. The particle refractive index and the particle absorption index were 1.5 and 0.1, respectively. 

### 2.3. Water Sorption Analysis

The sorption and desorption kinetics of water vapor applied to a sample of the investigated material were measured using a gravimetric fully automated Aquadyne DVS dynamic vapor sorption device (Quantachrome Instrumens, Hartley Wintney, UK). The measurements were performed at a constant temperature of 22.5 °C. Microcrystalline cellulose CRM 302 MCC was used as a standard for the device calibration. A sample of a defined weight of 45 mg was placed on analytical balance in the instrument, then a re-testing process was started, where the sample was cyclically exposed to changes in relative humidity and the dependence of the change in weight of the examined material on ambient humidity was measured. When the humidity limit is reached, the humidity increase cycle stops and the desorption process begins. The output of the measurement is the dependence between the change in the weight of the examined sample and the relative humidity for the adsorption and desorption process. The actual moisture of the powder was analyzed using a moisture analyzer MB 60 (VWR, Lutterworth, UK).

### 2.4. High Shear Granulation

Avicel PH101 was granulated by high shear wet granulation (HSWG) with a vertical, three- bladed bottom driven granulator of our own production [50]. The amount of Avicel per batch was 500 mL, i.e., 167 g. As the granular material was pure Avicel, it was not necessary to mix the material before the HSWG process. At the set chopper speed (1000 rpm) and impeller speeds (300, 500, and 700 rpm), granulation liquid was sprayed into the granulator at a flow rate of 22.5 mL/min. After the addition of the granulation liquid was completed, the whole wet mass was mixed for another 5 min, at the same impeller and chopper speeds. An appropriately set combination of impeller and chopper speeds ensures a balance, allowing establishment of good toroidal flow to promote granule growth [51]. The experiment was repeated twice, for the same duration of the granulation liquid adding. 

### 2.5. Classification of Franules

The wet granules from the HSWG process were weighed using a Nimbus NBL 254i (Adam Equipment, Kingstone, UK). After weighing, the wet granules were dried at 60 °C for 24 h. The dried granules were weighed again. The liquid to solid ratio (L/S ratio) parameter was determined as the difference between the weight of the wet and dry granulate divided by the weight of the dry granulate to accurately express the liquid content of the granules. The dried granules were classified as fine (<0.4 mm), yield (product) (0.4 up to 3 mm) and coarse (>3 mm) using sieve analysis by a Vibratory Shaker (Fritsch, Oberstein, Germany, with sieves of 0.4 mm and 3 mm. The individual fractions of the classified granules were weighed again. 

### 2.6. Measurement of Granule Size and Shape

**Figure 1:**
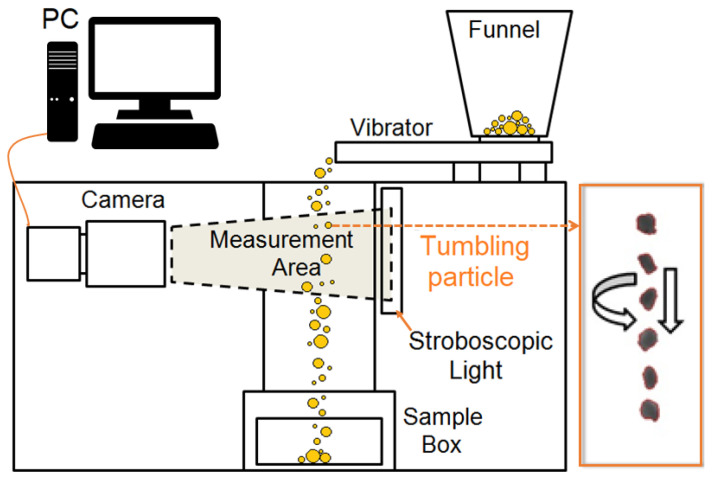
Principle of measurement by Dynamic image analysis (DIA) using PartAn 3D.

The size distribution and shape of the granules were analyzed using a PartAn 3D device (Microtrac MRB, Haan, Germany). The measurements were performed according to ISO 13322-2 and 9276-6 standards for dynamic image analysis [52]. Dynamic image analysis (DIA) is a technique that characterizes granules in motion by digitalizing photos of each granule from a CCD (charge-coupled device) camera (100 photos per second) and storing them in an image file. The particles tumble and rotate and are illuminated by stroboscopic light. The images are used to calculate morphological parameters based on the known size and location of the pixels in each image. The principle of measurement is shown in the figure (Figure 1). 

Both the granules and the powdered Avicel were evaluated volumetrically. The description of the size parameters is in Table 1. The characteristic dimension for determining the size of a granule was the Area Equivalent Diameter *D_a_* Equation (1) obtained from multiple photos of individual granules.

(1) $$D_{a}=\sqrt{4\;A/\pi \;},\;$$

where *A* is the area of the projected particle. Mean granule size was calculated according to the relationship in Equation (2).

(2) $$d_{\mathrm{mean}}=\frac{\sum \%\;in\;class\times mid.class\;size\;}{number\;of\;classes}/100.\;$$

Various shape parameters such as sphericity φ were used to determine the shape of the granulate Equation (3). Sphericity value φ = 1 corresponds to a perfect sphere.

(3) $$\varphi =\frac{D_{a}}{D_{p}},$$

where *D_p_* is the equivalent perimeter diameter calculated as (*p*/π). Another parameter is roundness *r* Equation (4). Value *r* = 1, corresponds to a perfectly circular particle.

(4) $$r=4\times \frac{A}{\pi }\times F_{L}^{2}\;,$$

where *F_L_* is the Feret length. The aspect ratio *AR* Equation (5) was defined as thickness to the particle length expressed by Feret parallel tangent dimensions. The value *AR* = 1 corresponds to a perfect sphere.

(5) $$AR=\frac{F_{T}}{F_{L}},$$

where *F_T_* is the Feret thickness. The largest Feret size measured in a series of individual particle images was assigned as the 3D length *F_L_* and the smallest Feret size was assigned as the thickness *F_T_* of that particle [52]. The last parameter describing the shape of the particles was concavity *c* Equation (6). The value *c* = 1 describes an extremely rough, spikey surface.

(6) $$c=CH_{A}-\frac{A}{CH_{A}},$$

where *CH_A_* is convex Hull area. In the same way as *d*_mean_, mean sphericity, roundness, aspect ratio and concavity were also determined as mean values from all granules classified as product in the whole batch.

### 2.7. Microscopic Analysis

The surface of the granules was analyzed using a JSM electron microscope (JEOL Ltd., Tokyo, Japan). The examined sample was sprayed with a layer of platinum nanoparticles before measurement in order to obtain a conductive surface. Next, the sample was placed in the working chamber of the microscope, where a vacuum was created. By the action of a beam with voltage 10.0 kV, the material was irradiated and the subsequent application of the secondary electron detector method (SED) revealed electrons reflected from the surface of the sample. 

### 2.8. Compressibility of Granules

A FT4 powder rheometer (Freeman Technology, Tewkesbury, UK) was used to determine the compressibility of Avicel powder and granules in accordance with ASTM-D7891-15 (2015) standard [53]. The batch of experimental material for one measurement was 60 g for Avicel powder and 80 g for granules. Compressibility measurements were performed in two vessels with a diameter of 50 mm and a volume of 85 mL. Prior to the compressibility measurement itself, the sample was subjected to three conditioning cycles, using a blade, in order to obtain a homogeneously packed powder bed. Excess material was split to achieve a defined bottom vessel volume 85 mL. Normal stress in a range of 0.5–15 kPa was applied to the pretreated sample. Based on the position of the piston, the height occupied by the test material before and after the compression test was determined and the volume of material was calculated using the vessel geometry and the measured heights. Compressibility (CPS) is calculated from the change in volume of the tested sample after compression, expressed as a percentage [54]. Despite the fact that compressibility is not a direct indicator of flow properties, it is related to several operations such as storage in hoppers or the behavior of bulk materials during roller compaction [55]. 

### 2.9. Data Processing

OriginPro 9.0 software (OriginLab Corporation, Northampton, MA, USA) was used to process the experimental data and box charts. Design Expert 13 (StatEase Inc., Minneapolis, MN, USA) was used for the creation of models. Response surface methodology with a quadratic model was used to correlate responses and factors (independent variables). The process parameters impeller speed and L/S ratio were chosen as independent variables. Parameters *d*_mean_ and process yield were chosen as the response (dependent variable). A general polynomial equation was used to create the model Equation (7)

(7) $$Y=\beta_{0}+\beta_{1}X_{1}+\beta_{2}X_{2}+\beta_{11}X_{1}^{2}+\beta_{22}X_{2}^{2}+\beta_{12}X_{1}X_{2},\;$$

where *Y* is a dependent variable (response), $\beta_{0}$ is a constant, $\beta_{1}$ and $\beta_{2}$ are linear coefficients, $\beta_{11}$ and $\beta_{22}$ are quadratic coefficients, $\beta_{12}$ is the interaction coefficient. *X*_1_ and *X*_2_ are the coded values of independent variables. Multilinear analysis and analysis of variance (ANOVA) were performed to established statistical significance [56]. 

## 3. Results and Discussion

### 3.1. Evaluation of Particle Size of Avicel PH 101

**Figure 2:**
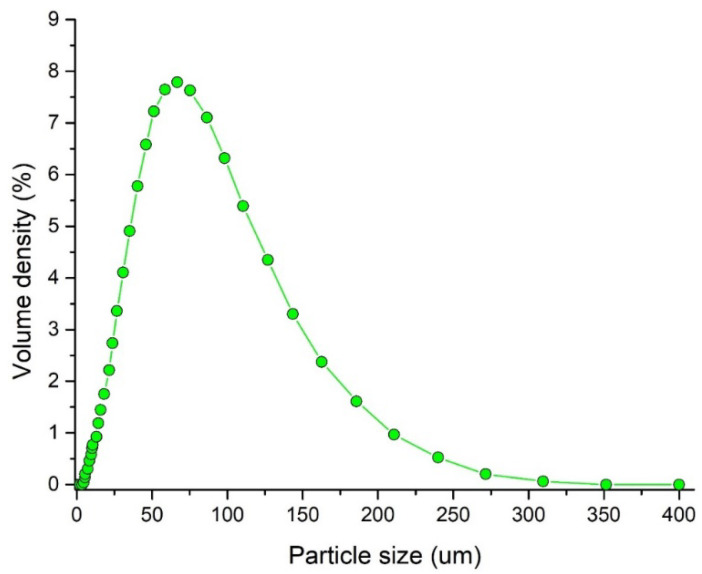
Particle size distribution of Avicel PH101 analyzed by Mastersizer 3000.

Values *d*_10_ (23.9 μm), *d*_50_ (64.2 μm) and *d*_90_ (140 μm) were determined by the dry cell method. These are the particle sizes corresponding to 10%, 50%, and 90% of the cumulative volume distribution curve. The distribution curve of the particles was unimodal (Figure 2). The span value of 1.807 calculated as (*d*_90_ − *d*_10_)/*d*_50_ and Sauter volume/surface diameter *d*_32_ of 46 μm were detected. The particle shape will be discussed later.

### 3.2. Evaluation of Water Sorption of Avicel PH 101

**Figure 3:**
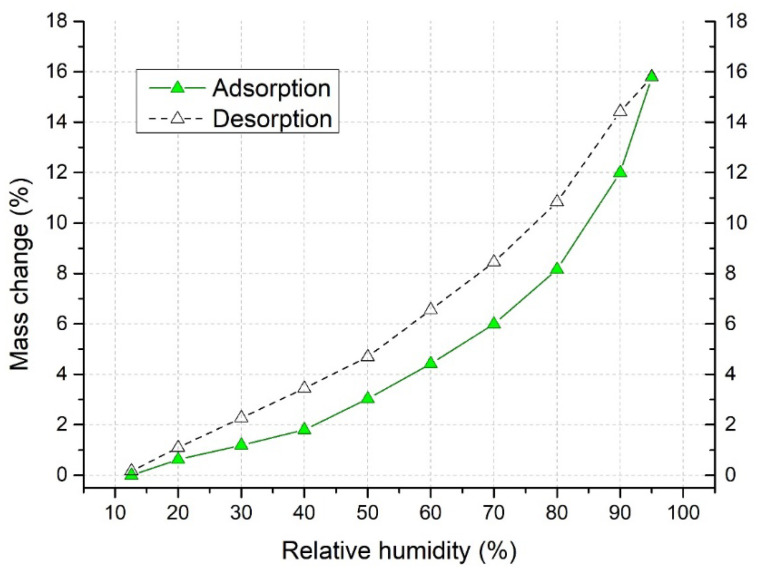
Adsorption and desorption isotherms of Avicel PH101 analyzed by Aquadyne DVS.

Avicel PH101 particles tend to increase in volume with increasing liquid content, which is desirable when tablets disintegrate. However, due to changes in relative humidity, Avicel PH101 particles can absorb significant amounts of moisture. Figure 3 shows the adsorption and desorption isotherms analyzed by Aquadyne DVS. At 95% relative humidity, the increase in the weight of the analyzed sample was up by 15.8%, meaning that the weight of the sample increased to 71.1 mg compared to the original 45 mg. The desorption cycle was able to dry the sample at a relative humidity of 12.6% to a value where the weight gain was 0.17%. Sun [57] pointed out that moisture trapped in Avicel particles can significantly influence its behavior during the compaction process. The moisture of our Avicel PH101 powder, analyzed by a MB 60 moisture analyzer, was 4.0 ± 0.2%.

### 3.3. Evaluation of Granule Size

**Figure 4:**
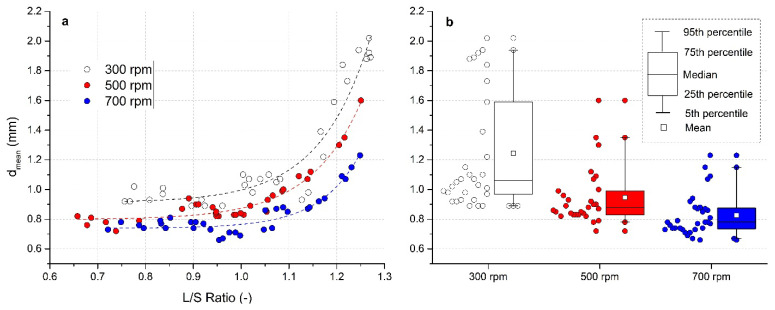
Evaluation of mean granule size for impeller speeds 300, 500, and 700 rpm: (**a**) effect of the L/S ratio on mean size *d*_mean_, (**b**) box charts of *d*_mean_ for different impeller speeds. The picture shows a description of box charts.

Graphical dependence of *d*_mean_ on the L/S ratio parameter is illustrated in Figure 4a for all impeller speeds. With increasing L/S, the *d*_mean_ of the formed granules increased almost linearly, up to the value L/S = 1.0. Within the L/S ratio 0.9–1.0, a “stagnant zone” (or the mild decrease in size) in the value of *d*_mean_ was noted. After exceeding the L/S ratio 1.0, the size of the granules increased exponentially, which indicates that the saturation point has been achieved and surpassed. The excess binder solution is then available for extensive granule growth by coalescence.

A similar trend can be found in the work by Shi et al. [58] during granulation of Avicel PH101 using distilled water and also in the work by Chitu et al. [59] during granulation of MCC with lactose. Osei-Yeboah et al. [60] found that at more than 45% water content, MCC–PVP granules flow well but cannot be compressed into intact tablets. Such changes in powder performance correspond to the rapid growth into large and dense spheres with a smooth surface. 

A significant effect of impeller speed on the mean granule sizes for all experiments is summarized in Figure 4b in a visual form of box charts. For better clarity, the points in the graph relating to individual experiments are included. In order to maintain a uniform range of L/S ratio, three experiments were excluded from the analyzes at L/S < 0.70 and impeller speed 500 rpm. In a numerical form, data of *d*_mean_ for different impeller speeds are presented in Table 2. 

The granules obtained at higher impeller speeds have a smaller particle size due to more pronounced dynamic stresses followed by subsequent breakage and attrition. The granules at an impeller speed of 300 rpm ranged in size from 0.89 to 2.02 mm. At an impeller speed of 500 rpm, the granules had a size of 0.72–1.6 mm, while the size of 0.66–1.23 mm was detected at 700 rpm. Rahmanian et al. [61] proved that granules formed at higher impeller speeds also have higher crushing strength due to lower porosity. 

The width of the granule size distribution decreased as the impeller speed increased; it was the narrowest at a speed of 700 rpm (Figure 4b). Mean granule size from all experiments, analyzed by box charts at an impeller speed 700 rpm was *d*_mean_ = 0.826 mm. At an impeller speed of 300 rpm it was up to 1.244 mm. Since some of the experiments are located in the area of L/S < 1, where the growth of the granules was not so significantly affected by the amount of added liquid, the median of the distribution curve was 1.06 mm (300 rpm), 0.88 mm (500 rpm), and 0.78 mm (700 rpm).

### 3.4. Evaluation of Process Yield

**Figure 5:**
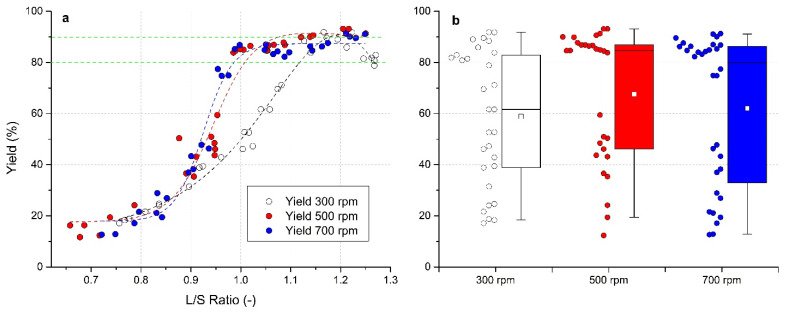
Evaluation of yield fraction (0.4–3 mm) for impeller speeds of 300, 500, and 700 rpm: (**a**) effect of the L/S ratio on yield fraction, green lines indicate yield interval from 80 to 90%, (**b**) box charts of yield fraction for different impeller speeds.

A graphical dependence between the process yield and the L/S ratio is shown in Figure 5a. With an increasing L/S ratio, the proportion of required granule fraction marked as yield (0.4 up to 3 mm) increased, however, the results were influenced by impeller speed as well. At an impeller speed of 300 rpm, the increase in yield is slower than at higher speeds and reached the value of 80% at the high L/S ratio of 1.12. With a further increase in L/S, the yield fraction increased to above 90%. The decrease in the curve at L/S > 1.25 shown in Figure 4a at 300 rpm was due to the removal of larger granules, which were classified as coarse. At impeller speeds of 500 and 700 rpm, contrarily, the content of yield granule portion raised sharply when exceeding the L/S ratio of 0.87 and significant changes in the process yield were observed particularly in the L/S range from 0.9 to 1.0. The 80% limit was achieved at a L/S ratio of approximately 0.97 at both speeds. After exceeding this value, the yield continued to grow slowly, achieving process yield >90% at a L/S ratio of 1.2 The graph shows that the highest process yields were achieved at higher L/S ratio values and higher impeller speeds. 

In Figure 5b, a summary box chart of the yield fraction distribution for each impeller speed are shown; in a numerical form, data are summarized in Table 2. The highest yield with the narrowest distribution was detected at an impeller speed of 500 rpm with the mean yield 67.6%. However, most experiments had a process yield above 80%, which was reflected in a median size yield of 84.6% at 500 rpm. At 700 rpm, the median was 79.8%. On the other hand, the lowest median yield value of 61.6% was noted in experiments at 300 rpm impeller speed.

### 3.5. Evaluation of Sphericity

**Figure 6:**
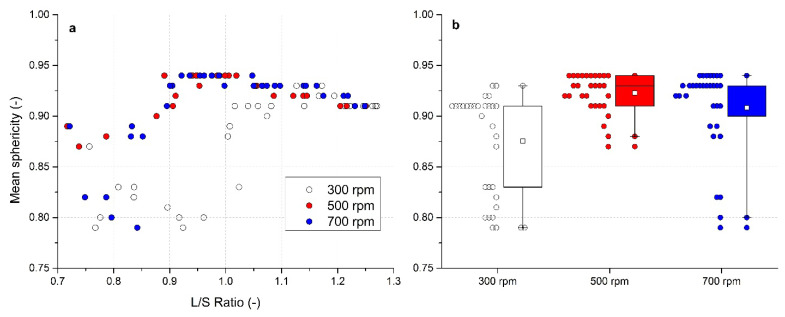
Evaluation of mean sphericity for impeller speeds of 300, 500 and 700 rpm: (**a**) effect of the L/S ratio on sphericity, (**b**) box charts of sphericity for different impeller speeds.

Spherical shape is a significant advantage for good flowability [62]. The relationship of mean sphericity φ of the formed granules on the L/S ratio and impeller speeds used was investigated and is illustrated in Figure 6a. At an impeller speed of 300 rpm, sphericity firstly decreased with increasing L/S ratio up to 1.0; then started to rise again. At L/S ratios > 1.0, almost all granule samples showed a sphericity value φ > 0.9. At the highest impeller speed of 700 rpm, a similar sphericity decrease at low L/S ratios was detected with the values of φ < 0.9 in the L/S ratio range of 0.7–0.85. Then, the granules showed higher values of φ (around 0.94) within the L/S ratio range of 0.92–1.04, decreasing mildly at higher L/S ratios, but still remaining above 0.9. The best results were noted at 500 rpm. The granules sphericity near to 0.9 (φ = 0.87) occurred even at low L/S. The value raised above 0.9 at L/S ratios higher than 0.87. Bouwman et al. [63] found that spherical and regularly shaped granules in the formulation containing MCC were formed particularly with a L/S ratio = 1.0. Our results at higher impeller speeds are in a good agreement with this observation.

In Figure 6b, a box chart of mean sphericity distributions for each impeller speed is presented; a numerical form is summarized in Table 2. The graph confirms the narrowest distribution of sphericity at 500 rpm. Mean sphericity values of 0.875 ± 0.049, 0.923 ± 0.019, and 0.908 ± 0.043 were noted at 300, 500, and 700 rpm, respectively. The median value of φ = 0.91 was observed at 300 rpm; at 500 and 700 rpm, the same value 0.93 was found. These median values from box chart analysis values were not so significantly affected by sphericity values at lower L/S ratios. Thus, it can be concluded that more spherical granules were formed at higher impeller speeds.

On the basis of sphericity, Maroof et al. [64] classified the particles as follows φ = 0.45 to 0.6—medium sphericity, φ = 0.6 to 0.8—spherical and φ = 0.8 to 1.0—high sphericity. According to this classification, most granules prepared in this work were categorized as highly spherical. 

### 3.6. Evaluation of Aspect Ratio

**Figure 7:**
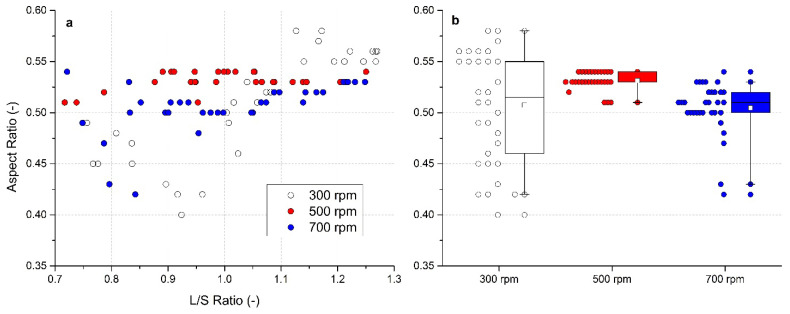
Evaluation of the aspect ratio for impeller speeds of 300, 500, and 700 rpm: (**a**) effect of the L/S ratio on aspect ratio, (**b**) box charts of aspect ratio for different impeller speeds.

Similarly to sphericity, the Aspect ratio *AR* parameter showed higher values with an increasing L/S ratio. The dependence between *AR* and L/S ratios is shown in Figure 7a. Out of all experimental runs, the granules formed at an impeller speed 300 rpm reached the highest values of *AR* as well as the highest data variability. The *AR* firstly decreased (minimum *AR* = 0.4 at L/S = 0.92), then it rose sharply with an increasing content of the liquid used to form the granules. The highest values were noted at L/S > 1.1 (maximum *AR* = 0.58). At the highest impeller speed 700 rpm, a decrease in *AR* values was observed at low L/S ratios (minimum 0.42 at L/S = 0.84) following with the *AR* increase to approximately 0.50–0.51 if the L/S ratio increased up to 1.05. Then, a further slight increasing trend of *AR* values at higher L/S was noted. At an impeller speed of 500 rpm, the most consistent data were detected; all granules had the *AR* values of at least 0.51 regardless of the L/S ratio used. The higher *AR* values indicate better flow properties [65]. 

Again, an *AR* distribution box chart for each impeller speed can be seen in Figure 7b and in numerical form in Table 2. The graph clearly documents that the narrowest *AR* distribution was noted at 500 rpm impeller speed. The mean value of *AR* was 0.507 ± 0.054 (300 rpm), 0.531 ± 0.009 (500 rpm) and 0.505 ± 0.026 at 700 rpm. At 500 rpm, the median value (0.53) of the *AR* distribution was almost identical to the mean value. The median value of 0.515 at 300 rpm was almost identical to that of 0.51 at 700 rpm. 

Based on the sphericity and *AR* values, it can be concluded that most of the granules prepared in all experimental runs had an elongated rounded shape.

### 3.7. Evaluation of Roundness

**Figure 8:**
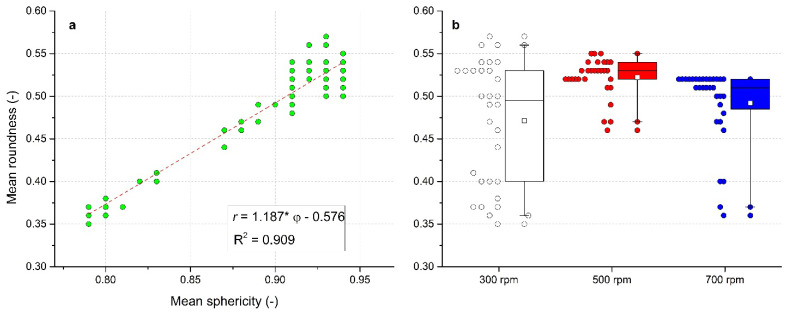
Evaluation of mean roundness. (**a**) Mean roundness relationship on mean sphericity, (**b**) box charts of mean roundness for impeller speeds 300, 500, and 700 rpm.

Roundness *r* represents another granulometric parameter. When analyzing the experimental data, a similar trend to that described above for sphericity was found in relationship to the L/S ratio and impeller speed. The linear relationship between mean *r* and mean φ illustrated in Figure 8a for all experimental data can be described by a linear regression *r* = 1.187 × φ − 0.576 with the coefficient of determination R^2^ = 0.909 (*p* < 0.005). 

In a box chart of roundness distribution at each impeller speed (Figure 8b), the narrowest *r* distribution with the rounded granules at 500 rpm is demonstrated. Similarly to the evaluation of φ and *AR*, the highest values of *r* = 0.57 to 0.58 (L/S = 1.12 to 1.17) as well as the widest distribution were obtained at 300 rpm impeller speed; low values of *r* were found up to L/S = 1. Finally, the lowest *r* values were noted for four granules batches formed at lower L/S values at 700 rpm.

According to the classification by Maroof et al. [64], values *r* = 0.35 to 0.49 represent sub-rounded particles, *r* = 0.49 to 0.7 belong to rounded particles, and *r* = 0.7 to 1.0 belong to well-rounded particles. The median values of *r* = 0.495 (300 rpm), 0.53 (500 rpm), and 0.51 (700 rpm) were registered in this work. Mean values from box chart for each impeller speed were 0.471 ± 0.072 (300 rpm), 0.522 ± 0.023 (500 rpm), and 0.492 ± 0.046 (700 rpm). Based on the abovementioned classification, most prepared granules can be characterized as rounded particles. In a numerical form, data are also summarized in Table 2.

### 3.8. Evaluation of Roughness

**Figure 9:**
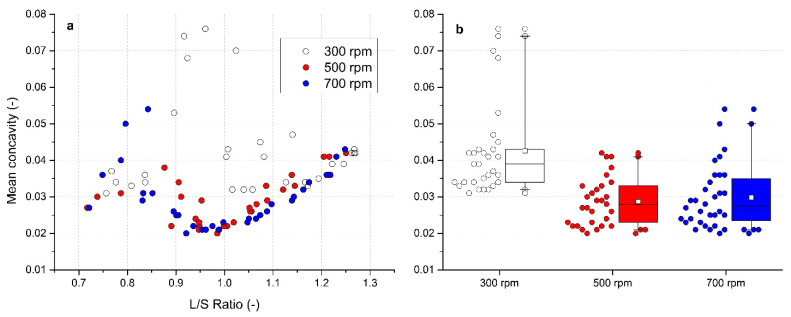
Evaluation of mean concavity for impeller speeds 300, 500, and 700 rpm: (**a**) effect of the L/S ratio on mean concavity, (**b**) box charts of mean concavity for different impeller speeds.

The roughness of the granules was described by concavity *c*. A graphical dependence of mean *c* on the L/S ratio in Figure 9a shows that the roughest granules were formed at the lowest impeller speed of 300 rpm. In a range of L/S ratio 0.9–1.0, the values of *c* were even larger than 0.07. In opposite, the most homogeneous data were noted at 500 rpm again. At higher impeller speeds, there was first a rising roughness trend at L/S = 0.7 to 0.8, which then descended at L/S = 0.8–0.95. Starting at an L/S ratio of approximately 0.95, a linear increase in *c* value in regard to the increase in L/S value was visible at both 500 rpm and 700 rpm. Lower values of roughness have a positive effect on the flow properties of powder materials and granules [66]. Based on the results, the smoothest granules were formed at higher impeller speeds in the L/S range of 0.9 to 1.05. 

A summary of the mean concavity for all experiments in a visual form of box charts of *c* for all impeller speeds is in Figure 9b and in numerical form in Table 2**.** At an impeller speed of 300 rpm, the highest mean concavity was observed (0.042 ± 0.013; median = 0.039). The mean concavity values as well as the median values were comparable (0.029 and 0.028, respectively) at an impeller speed of 500 rpm and 700 rpm. However, the graph confirms the narrowest distribution of *c* in the investigated range of the L/S ratio at an impeller speed of 500 rpm.

### 3.9. Effect of Granule Size on Shape Factors

**Figure 10:**
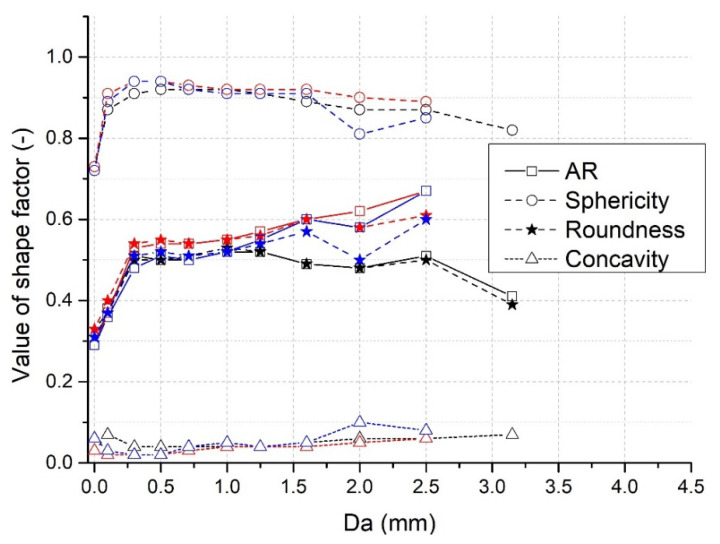
Relationship of investigated shape factors on area equivalent diameter *D_a_* at the L/S ratio = 1.0 at impeller speeds of 300 (black), 500 (red), and 700 rpm (blue).

As discussed above, granulometric parameters of the produced granules were influenced by the L/S ratio and the impeller speed used. Apart from these process parameters, the actual values of shape factors depended also on the actual size of the individual granules in the batch. The graphical dependence of investigated shape factors on the area equivalent diameter *D_a_* at the L/S ratio = 1.0 is summarized in Figure 10.

The graph shows that sphericity φ reaches the smallest value for granules up to 0.1 mm. The highest values of φ = 0.94 were detected for granules in the range of 0.3–0.5 mm at 500 and 700 rpm. When the area equivalent diameter *D_a_* increased further, a slight decrease in the values of φ regardless of the impeller speed was noted. The lowest values of aspect ratio *AR* were also detected for the smallest granules, but they increased quickly with the increase in the *D_a_* up to 0.3 mm at all impeller speeds used. Then at the impeller speed of 300 rpm, *AR* remained near to 0.5 up to approximately 2.5 mm granules and decreased with larger granules. At impeller speeds of 500 and 700 rpm, slightly higher invariable values of *AR* (around 0.55) were noted, up to approximately 1.0 mm granules, which then increased with the area equivalent diameter *D_a_*. The highest *AR* values were reached at the 500-rpm impeller speed. As clearly illustrated in Figure 10, roundness *r* is narrowly associated with the *AR* showing the same trend. The exclusion is observable only with the granules larger than 2 mm at higher impeller speeds. 

Even though the higher values of concavity *c* were particularly registered for the smallest granules at 300 rpm, the low oscillating values were noted later up to the granule size 1.5 mm regardless of impeller speed. Then, they increased with the further increase in *D_a_*, achieving the highest value *c* = 0.1 at granule size 2.0 mm at 500 rpm. 

**Figure 11:**
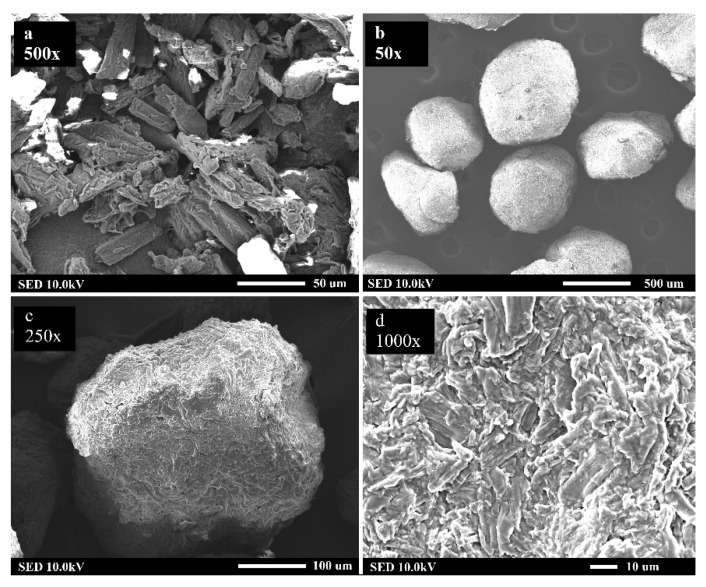
SED images of granules from electron microscope: (**a**) raw particles of Avicel PH101 at 500× magnification, (**b**) granules produced at L/S ≈ 1.0 and 700 rpm impeller speed at 50× magnification, (**c**) surface of granule at 250× magnification, (**d**) detail of granule surface at 1000× magnification.

The raw particles of Avicel PH101 were analyzed by electron microscopy using the SED method at 500× magnification; the rod-shaped particles are shown in Figure 11a. In order to illustrate granulometric parameters of the prepared granules, those formed at L/S ratio = 1.0 and an impeller speed of 700 rpm are chosen. Figure 11b shows the smooth surface, rounded spherically shaped (φ = 0.94) granules. It can be seen that the characteristic dimensions of the granules are not the same in all directions, which confirms the lower *AR* values. A more detailed view of the granule from this batch can be found in Figure 11c, where asperities (*c* = 0.022) on the surface at 250× magnification are visible. In detail, the raw rod-shaped particles of Avicel PH101 can be recognized on the surface of the granules in Figure 11d. A similar morphology of granules can be found in the work of Mahdi et al. [67]. Shi et al. [68] proved that the final shape of the granules can also be influenced by the process time of wet massing. 

### 3.10. Evaluation of Granules Compressibility 

Particles with lower compressibility are generally advantageous for the tableting process due to more uniform filling of the die and less frequent troubles during transport [69]. The compressibility value (CPS) obtained from the FT4 rheometer for Avicel powder was high (15.69 ± 0.48%), showing its poor flow properties. Contrary, all granules produced within the wide range of L/S ratio at three different impeller speeds showed lower values of CPS.

**Figure 12:**
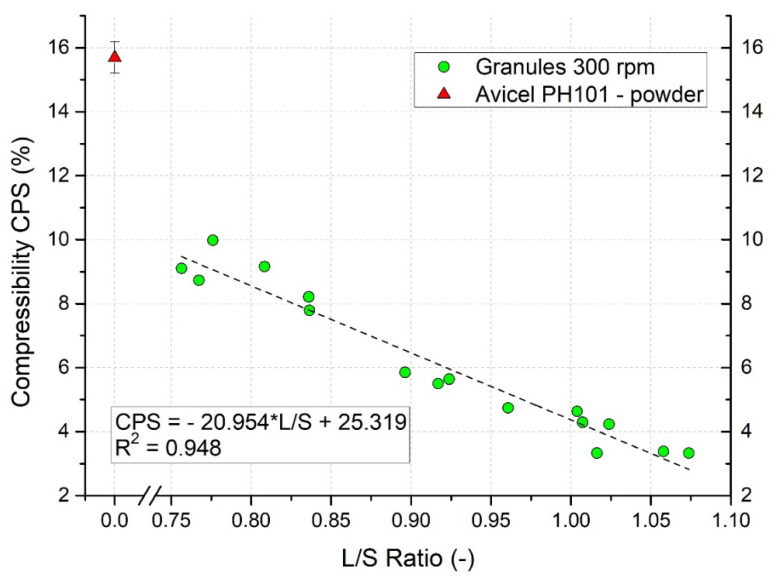
Compressibility of raw Avicel PH101 powder and effect of L/S ratio on compressibility of granules manufactured at 300 rpm impeller speed.

The effect of L/S ratio on the compressibility of granules manufactured at 300 rpm impeller speed is present in Figure 12. As discussed above, these granules were the biggest, having the lowest sphericity and high concavity. A decrease in the compressibility value with an increase in L/S ratio was observed; with a higher liquid content, the formed granules had the higher density, which was reflected in the CPS value. CPS could be analyzed up to the L/S ratio 1.07 when the value of CPS = 3.33%. For granules formed at the higher L/S ratio, the device showed an overload at 15 kPa and automatically terminated the measurement. 

The relationship between CPS (%) and L/S ratio for 300 rpm impeller speed was described by linear regression of the experimental data: CPS = 25.319 − 20.954 × L/S, with the coefficient of determination R^2^ = 0.948. This clearly demonstrates that CPS value can be efficiently predicted by L/S ratio.

### 3.11. Application of Surface Response Methodology to Predict Granule Size and Process Yield

A general polynomial equation Equation (7) was used. Based on experimental data, models for determining *d*_mean_ and process yield (dependent variables), were created using Design Expert 13 software depending on the impeller speed (IS) and the L/S ratio (independent variables). The relationship was described by equations Equations (8) and (9).

(8) $$d_{\mathrm{mean}}=3.22765-6.10009(L/S)+0.000907\left(\mathrm{IS}\right)-0.002795\left(L/S\times \mathrm{IS}\right)+4.41496{\left(L/S\right)}^{2}+1.0458\times 10^{-6}{\left(\mathrm{IS}\right)}^{2}\;$$

(9) $$\mathrm{Yield}=425.81688(L/S)+0.228893\left(IS\right)+0.056508\left(L/S\times IS\right)-146.81044{\left(L/S\right)}^{2}-0.000257{\left(IS\right)}^{2}-286.33147\;$$

**Figure 13:**
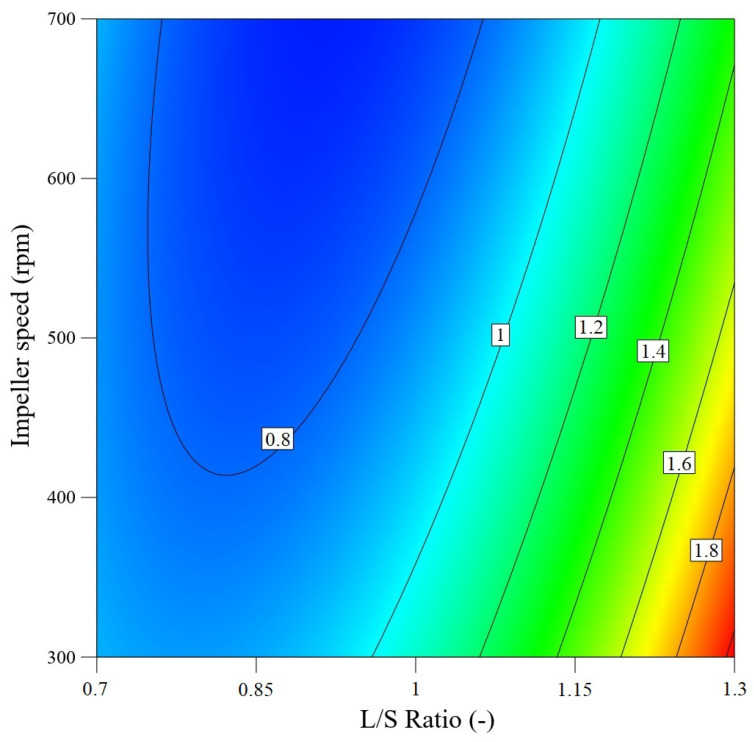
Contour plot of quadratic model for prediction of mean granule size as function of L/S ratio and impeller speed.

A contour plot model for prediction of *d*_mean_ in relationship to the L/S ratio and the impeller speed is shown in Figure 13**.** A significant area with *d*_mean_ = 0.8 mm (blue), which occupies more than half of the graph was detected. The increase in L/S ratio and the associated increase in the size of the granules formed is shown by the change in color. At lower impeller speeds, an orange to red area shows the largest formed granules depending on the L/S ratio. Based on the graph, the process parameters can be optimally set in order to obtain granules of a defined size. Using this, the prediction of *d*_mean_ value with a standard deviation of 0.077 mm is possible. 

**Figure 14:**
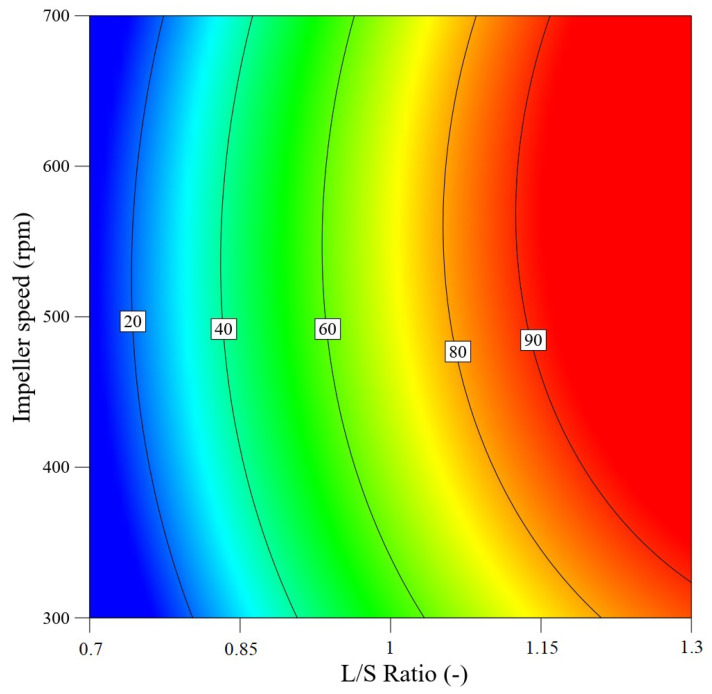
Contour plot of the quadratic model for prediction of the process yield as a function of L/S ratio and impeller speed.

Similarly, the experimental data was processed in order to predict the process yield; the model is shown in Figure 14. In this graph, the transition between the individual areas of the process yield is significantly smoother. A significant part of the graph is the area with a yield of more than 80% shown in orange to red color. As a result, the process parameters of the impeller speed and L/S ratio can be optimally set in order to obtain a defined process yield.

The parameters of the analysis of variance (ANOVA) for both models can be found in Table 3 and Table 4. Values of *p* < 0.05 indicate that the parameters evaluated are statistically significant (>95% probability that the hypothesis is true). The highest *p* value of 0.0169 in *d*_mean_ model was detected with the independent variable IS^2^, which indicates its lowest significance. The model determination coefficient was R^2^ = 0.9436 with *p* < 0.0001. 

In the yield model, the highest *p* value of 0.1596 was detected with the independent variable L/S × IS. Similarly to *d*_mean_ model, the value of 0.0169 was noted for IS^2^. The determination coefficient for yield model was slightly lower R^2^ = 0.8905 with *p* < 0.0001.

**Figure 15:**
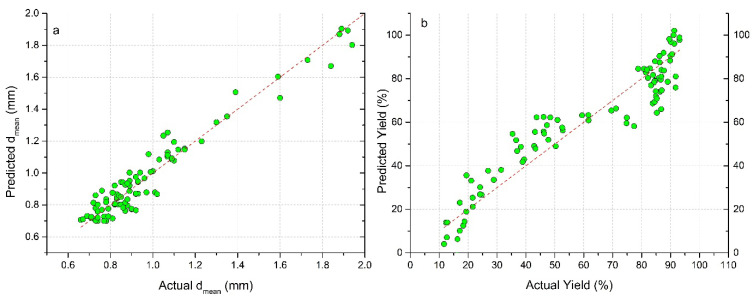
Relationships between experimental and predicted data: (**a**) model for prediction of mean granule size, (**b**) model for prediction of process yield.

The comparison between the experimental and predicted data for both models can be found in Figure 15a,b. The better prediction efficiency was obtained for mean granule size. 

## 4. Conclusions

The morphology of the granules based on microcrystalline cellulose and the yield of the process were analyzed in this systematic study with respect to the process parameters of high shear wet granulation. The granulometric parameters of the formed granules were analyzed by the dynamic image analysis by PartAn 3D as well as by the electron microscopy. The amount of added granulation liquid expressed by the liquid to solid ratio (L/S) in a range of 0.7 to 1.27 had a significant effect on all the monitored parameters: sphericity, roundness, aspect ratio, and concavity. Three different impeller speeds, 300 rpm, 500 rpm, and 700 rpm, were used. For the comparison reason, the same time of wet massing was employed.

Even a small change in the liquid content caused a significant change in the shape of the granules. At higher impeller speeds and with a L/S ratio ≈1, the most spherical granules with the smoothest surface were formed. In opposite, the higher dynamic stress at the highest impeller speed caused mechanical breakage and attrition, which led to the formation of granules of the smallest particle size. In terms of process yield and particle shape, the best granules were formed at an impeller speed of 500 rpm. The highest process yields were achieved at higher L/S ratio values and higher impeller speeds. Moreover, the values of the granulometric factors observed experimentally were influenced also by the size (area equivalent diameter) of the individual granules in the batch. Additionally, the linear relationship with the coefficient of determination R^2^ = 0.948 was detected between the amount of added granulation liquid and the compressibility of the granules (FT4 rheometer), allowing the efficient prediction of CPS by L/S ratio. By using response surface methodology, two separate models predicting either the size of granules or the process yield were obtained based on experimental data from more than 90 experiments. According to the amount of liquid added and the impeller speed, both proposed models allow the determination of the granule size or process yield with a high level of accuracy expressed by the coefficient of determination values R^2^ = 0.9436 and R^2^ = 0.8905, respectively. 

## Figures and Tables

**Table 1:** Description of size parameters used in 3D Dynamic image analysis.

Parameter	Symbol	Description	Scheme
Area	*A*	Area of projected particle. Calculated as an average area of the sequence of 3D images.	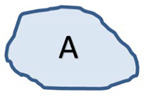
Perimeter	*P*	Perimeter of the projected image. Calculated as an average perimeter of the sequence of 3D images.	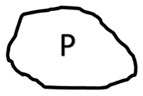
Convex Hull Area	*CH_A_*	The convex outline of a projected shape having concavities.	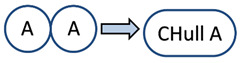
Area Equivalent diameter	*D_a_*	Spheres of equal area to the original particle. Average area in the sequence of 3D images.	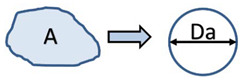
Equivalent Perimeter diameter	*D_p_*	Spheres of equal perimeter to the original particle. Average area in the sequence of 3D images.	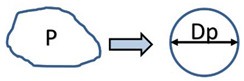
Feret length	*F_L_*	Maximal distance between parallel tangents.	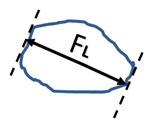
Feret thickness	*F_T_*	Minimum distance between parallel tangents. Measured as minimum particle width in the sequence of 3D images.	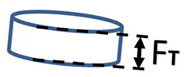

**Table 2:** Results of box charts analysis of individual investigated granulometric parameters and process yield.

	Impeller Speed (rpm)	Mean Value	STDEV	5th Percentile	25th Percentile	Median	75th Percentile	95th Percentile
*d*_mean_ (mm)	300	1.244	0.392	0.89	0.97	1.06	1.59	1.94
	500	0.946	0.192	0.78	0.83	0.88	0.99	1.35
	700	0.826	0.139	0.67	0.735	0.78	0.875	1.15
Yield (%)	300	58.9	26.2	18.3	38.8	61.6	82.8	91.8
	500	67.6	25.9	19.4	46.1	84.6	86.8	93.1
	700	62.0	29.2	12.8	32.9	79.8	86.2	91.0
φ (-)	300	0.875	0.049	0.79	0.83	0.91	0.91	0.93
	500	0.923	0.019	0.88	0.91	0.93	0.94	0.94
	700	0.908	0.043	0.8	0.9	0.93	0.93	0.94
*AR* (-)	300	0.507	0.054	0.42	0.46	0.515	0.55	0.58
	500	0.531	0.009	0.51	0.53	0.53	0.54	0.54
	700	0.505	0.026	0.43	0.5	0.51	0.52	0.53
*r* (-)	300	0.471	0.072	0.36	0.4	0.495	0.53	0.56
	500	0.522	0.023	0.47	0.52	0.53	0.54	0.55
	700	0.492	0.046	0.37	0.485	0.51	0.52	0.52
*c* (-)	300	0.042	0.013	0.032	0.034	0.039	0.043	0.074
	500	0.029	0.006	0.021	0.023	0.028	0.033	0.041
	700	0.029	0.008	0.021	0.024	0.028	0.035	0.05

**Table 3:** Analysis of variance for the *d*_mean_ model and individual variables.

	Sum of Squares	*df*	Mean Square	*F*-Value	*p*-Value
Model	8.49	5	1.70	284.41	<0.0001
L/S	2.17	1	2.17	363.35	<0.0001
IS	1.16	1	1.16	194.55	<0.0001
L/S × IS	0.4514	1	0.4514	75.60	<0.0001
L/S^2^	1.31	1	1.31	218.61	<0.0001
IS^2^	0.0354	1	0.0354	5.93	0.0169

**Table 4:** Analysis of variance for the process yield model and individual variables.

	Sum of Squares	*df*	Mean Square	*F*-Value	*p*-Value
Model	63,398.24	5	12,679.65	138.32	<0.0001
L/S	61,713.25	1	61,713.25	673.21	<0.0001
IS	1435.83	1	1435.83	15.66	0.0002
L/S × IS	184.50	1	184.50	2.01	0.1596
L/S^2^	1443.32	1	1443.32	15.74	0.0002
IS^2^	2144.73	1	2144.73	23.40	0.0169

## Data Availability

The data presented in this study are available on request from the corresponding author. The data are not publicly available due to extensive quantity of values.
